# KMnF_3_:Yb^3+^,Er^3+^ Core-Active-Shell Nanoparticles with Broadband Down-Shifting Luminescence at 1.5 μm for Polymer-Based Waveguide Amplifiers

**DOI:** 10.3390/nano9030463

**Published:** 2019-03-20

**Authors:** Yongling Zhang, Peng Lv, Dongxia Wang, Zhengkun Qin, Fei Wang, Daming Zhang, Dan Zhao, Guanshi Qin, Weiping Qin

**Affiliations:** 1 College of Information &Technology, Jilin Normal University, Siping 136000, China; lvpengjlnu@163.com (P.L.); wangdongxiajlnu@163.com (D.W.); qin_zhengkun@126.com (Z.Q.); 2 College of Electronic Science & Engineering, Jilin University, Changchun 130012, China; wang_fei@jlu.edu.cn (F.W.); zhangdm@jlu.edu.cn (D.Z.); dzhao@jlu.edu.cn (D.Z.)

**Keywords:** KMnF_3_:Yb^3+^,Er^3+^ core-shell nanoparticles, broadband, down-shifting luminescence, 1.5 µm, polymer-based waveguide amplifiers

## Abstract

In this study, we prepared cubic-phase oleic-acid-coated KMnF_3_: Yb^3+^,Er^3+^ nanoparticles (NPs) and NaYF_4_:Yb^3+^,Er^3+^ NPs, which were about 23 nm. From the down-shifting emissions spectra of the two NPs obtained by 980 nm excitation, we observed the fact that the KMnF_3_: 18%Yb^3+^,1%Er^3+^ NPs were a luminescent material with a broadband near-infrared emission of 1.5 μm, and full-width at half-maximum (FWHM) of 55 cm^−1^, which was wider than that of the NaYF_4_: 18%Yb^3+^,1% NPs. Therefore, we believe that the oleic-acid-coated KMnF_3_:Yb^3+^,Er^3+^ NPs have great potential in fabricating broadband waveguide amplifiers. Through epitaxial growth of a KMnF_3_: Yb^3+^ active-shell on the core NPs, we compounded KMnF_3_:Yb^3+^,Er^3+^@KMnF_3_:Yb^3+^ core-active-shell NPs whose 1.5-μm infrared emissions intensity was 3.4 times as strong as that of the core NPs. In addition, we manufactured waveguide amplifiers using KMnF_3_:18%Yb^3+^,1%Er^3+^@KMnF_3_:2%Yb^3+^ NPs as the core materials of the waveguide amplifiers. When the input signal power was 0.2 mW and the pump power was 200 mW, we achieved a relative gain of 0.6 dB at 1534 nm in a 10-mm long waveguide.

## 1. Introduction

Recently, erbium (Er^3+^)-doped waveguide amplifiers (EDWAs) have aroused a lot of research [1,2,3,4,5]. It is mainly due to two reasons: one is that Er^3+^ ions can send near-infrared emissions at around 1.5 μm (the ^4^I_13/2_ → ^4^I_15/2_ transition of Er^3+^ ions), the other is that EDWAs have great potential for application in the field of near-infrared optical communication technology [6,7,8]. Usually, EDWAs includes inorganic material waveguide amplifiers and polymer material waveguide amplifiers. Compared with inorganic material waveguide amplifiers using glasses [9,10,11] and oxide [12,13,14] as gain material, polymer material waveguide amplifiers have many advantages, such as low fabrication costs, simple processing, and easy integration with silicon [15,16,17]. Both Er^3+^-doped organic complexes [18,19] and Er^3+^-doped fluoride nanoparticles (NPs) [20,21] can be used to manufacture polymer material waveguide amplifiers. Compared with Er^3+^-doped organic complexes, Er^3+^-doped NPs have some advantages, for example high photostability [22,23].

However, it is difficult to achieve an Er^3+^-doped NP with a broadband emissions around 1.5 μm owing to the f–f transition of Er^3+^ ions [22]. So far, there have been many studies on the fluorescence properties of Er^3+^-doped NPs [24,25,26], but few works about the broadband near-infrared emissions (at around 1.5 μm) of Er^3+^-doped NPs. In this paper, we compounded KMnF_3_:18%Yb^3+^,1%Er^3+^ NPs with a broadband near-infrared emission. We analyzed the crystal structure and morphology of these NPs and measured the down-shifting luminescence properties. Then, we describe a method to improve the down-shifting luminescence intensity (at 1.5 µm) of KMnF_3_:18%Yb^3+^,1%Er^3+^ NPs by growing a KMnF_3_:2%Yb^3+^ active-shell. Finally, we fabricate waveguide amplifiers using KMnF_3_:18%Yb^3+^,1%Er^3+^@KMnF_3_:2%Yb^3+^ NPs as the gain medium. With an input signal power of 0.2 mW and a pump power of 200 mW, a relative optical gain of 0.6 dB at 1534 nm was obtained.

The main characteristics of this article are follows:

(1) We prepared KMnF_3_:18%Yb^3+^,1%Er^3+^ NPs with a broadband 1.5-μm emission. Its full-width at half-maximum (FWHM) was about 290 cm^−1^ and was 55 cm^−1^ wider than that of NaYF_4_:18%Yb^3+^,1% NPs with the same size and phase.
(2) We obtained KMnF_3_:18%Yb^3+^,1%Er^3+^@KMnF_3_:2%Yb^3+^ core-active-shell NPs with the strong and broadband 1.5-μm emission by coating a KMnF_3_:2%Yb^3+^ shell.

## 2. Experimental

### 2.1. Chemicals

All rare-earth nitrates Re (NO_3_)_3_·6H_2_O and rare-earth chloride ReCl_3_·6H_2_O we used came from Tianyi New Material, Jining, China. 1-octadecene (ODE) and oleic acid (OA) in the experiment came from the Alfa Aesar Company, Shanghai, China. The other chemical reagents were produced by Sinopharm Chemical Reagent, Beijing, China.

### 2.2. Synthetic Procedures

Synthesis of NaYF_4_:18%Yb^3+^,1%Er^3+^ NPs was conducted with a high-boiling solvent method which used the following steps [27].

The following chemical reagents: 0.01 mmol ErCl_3_·6H_2_O, 0.18 mmol ErCl_3_·6H_2_O, and 0.81 mmol ErCl_3_·6H_2_O were added into a 100-mL four-necked flask containing 6 mL OA and 15 mL ODE. The solution was heated to 150 °C and kept for 30 min under the protection of an Ar gas flow to remove residual O_2_ and water. The solution was naturally cooled to room temperature. A 10-mL methanol solution containing 0.15 g NH_4_F and 0.1 g NaOH was slowly added into in the 100-mL four-necked flask. Then, the reaction solution was heated to 50 °C for 30 min under stirring. Finally, the solution was heated to 280 °C and kept for 60 min. The solution was naturally cooled to room temperature, and cleaned by cyclohexane and ethanol four times.

Synthesis of the KMnF_3_:18%Yb^3+^,1%Er^3+^ core NPs by a solvothermal method had the following steps [28,29].

The following chemical reagents: 12 mmol KOH, 5 mL deionized water, 5 mL ethanol, and 10 mL OA were added into a 50-mL beaker. The reaction solution was stirred for 30 min at room temperature. While stirring, 0.072 mmol Yb (NO_3_)_3_·6H_2_O, 0.004 mmol Er (NO_3_)_3_·6H_2_O, and 0.324 mmol MnCl_2_ were added to the solution. After that, 3.5 mmol KF was added to the solution and the solution was stirred continuously for 30 min. Finally, the solution was transferred into a 50-mL reaction kettle and heated to 200 °C for 1 h. The solution of KMnF_3_:Yb^3+^,Er^3+^ NPs can be used to synthetize the core-shell NPs. The samples for measuring fluorescence spectra were washed by cyclohexane and ethanol four times.

Synthesis of the KMnF_3_:18%Yb^3+^,1%Er^3+^@KMnF_3_:2%Yb^3+^ core-active-shell NPs by a solvothermal method had following steps [29,30].

The following chemical reagents: 12 mmol KOH, 5 mL deionized water, 5 mL ethanol, and 10 mL OA were added into a 100-mL beaker. The reaction solution was stirred at room temperature for 30 min. Then, 0.008 mmol Yb (NO_3_)_3_·6H_2_O and 0.392 mmol MnCl_2_ were added into the solution. The solution of 0.4 mmol KMnF_3_:Yb^3+^,Er^3+^ NPs was added into the beaker. The reaction solution was stirred continuously for 30 min. While stirring, 3.5 mmol KF was added into the reaction solution and the solution was stirred continuously for another 30 min. Finally, the reaction solution was transferred into two 50-mL reaction kettles and heated to 200 °C for 12 h. These products were washed with ethanol and cyclohexane four times.

Fabrication processes of the KMnF_3_:18%Yb^3+^,1%Er^3+^@KMnF_3_:2%Yb^3+^ core-active-shell NPs doped polymer waveguides were used the following steps [5,31].

Step 1: form a PMMA (polymethyl methacrylate) film-base cladding layer by spin-coating PMMA polymers on silicon substrate-based thick silicon dioxide layer, and bake the silicon substrates for 2 h at 120 °C. Step 2: an inductively coupled plasma (ICP) etching approach and classical photolithography are used to form waveguide patterns on the PMMA film-base cladding layer. Step 3: form the core waveguides by spin-coating the mixture of KMnF_3_:18%Yb^3+^,1%Er^3+^@KMnF_3_:2%Yb^3+^ core-active-shell NPs and PMMA into the grooves, and cure it at 100 °C for 2.5 h. Finally, a PMMA polymer is spin-coated upon the core waveguides layer. The fabrication processes is shown in Supplementary Materials Figure S1.

### 2.3. Characterization

The crystalline phase of as-synthesized NPs was characterized by a Model Rigaku Ru-200b (Rigaku, Tokyo, Japan), when λ = 1.5406 Å and scanning range was 10–70°. The transmission electron microscope (TEM) image of as-synthesized NPs was recorded by a H-600 electron microscope under the condition of 200 kV (Hitachi, Tokyo, Japan). Under a 980 nm excitation, the fluorescence spectrum of as-synthesized NPs was recorded by a SPEX 1000M spectrometer (Horiba Group, Kyoto, Japan). The Fourier transform infrared spectrum of the as-synthesized NPs was characterized by a FTIR-1500 (Josvok, Tianjin, China), under the scanning range of 1000 cm^−^^1^–4000 cm^−^^1^. The scanning electron microscopy (SEM) image of the waveguide was characterized by a JSM-7500F (Jeol, Tokyo, Japan).

## 3. Results and Discussion

### 3.1. Crystal Structure and Morphology

**Figure 1:**
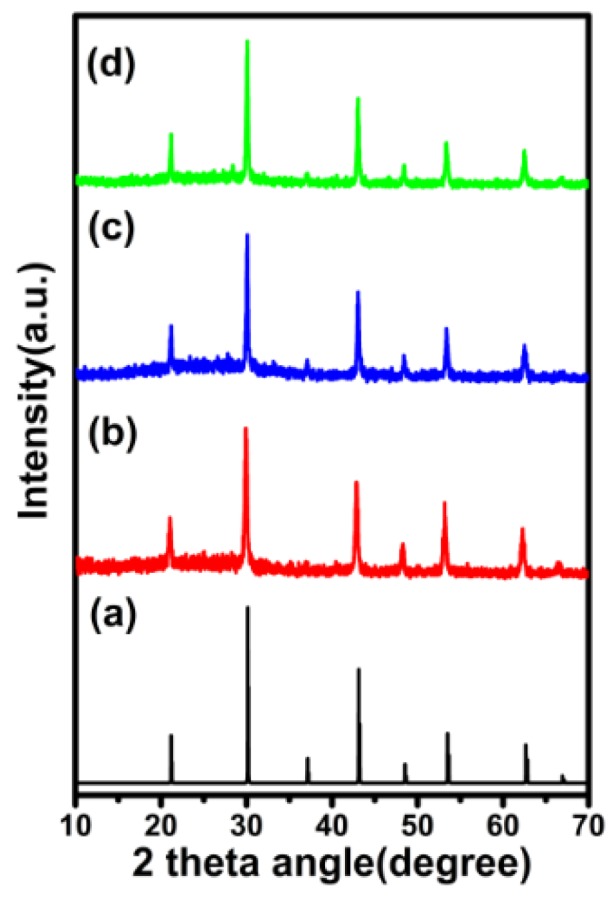
XRD patterns of (**a**) standard KMnF_3_ (JCPDS-82-1334), (**b**) KMnF_3_:18%Yb^3+^,1%Er^3+^ nanoparticles (NPs), (**c**) KMnF_3_:18%Yb^3+^,1%Er^3+^@ KMnF_3_ NPs, and (**d**) KMnF_3_:18%Yb^3+^,1%Er^3+^@KMnF_3_:2%Yb^3+^ NPs.

**Figure 2:**
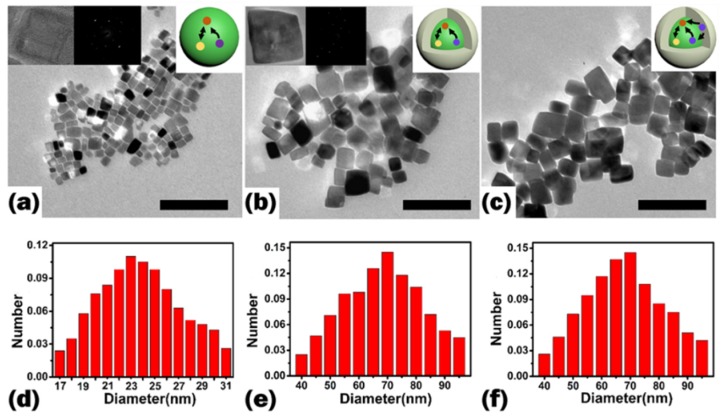
TEM images of XRD patterns of (**a**) KMnF_3_:18%Yb^3+^,1%Er^3+^ NPs, (**b**) KMnF_3_:18%Yb^3+^, 1%Er^3+^@ KMnF_3_ NPs, (**c**) KMnF_3_:18%Yb^3+^,1%Er^3+^@ KMnF_3_:2%Yb^3+^ NPs (the inset shows a schematic illustration of corresponding NPs). Histograms of (**d**) KMnF_3_:18%Yb^3+^,1%Er^3+^ NPs, (**e**) KMnF_3_:18%Yb^3+^,1%Er^3+^@ KMnF_3_ NPs, and (**f**) KMnF_3_:18%Yb^3+^,1%Er^3+^@KMnF_3_:2%Yb^3+^ NPs size distributiond obtained from TEM. The scale bar is 200 nm in (**a**), (**b**), and (**c**).

In order to get the crystal structure of as-synthesized KMnF_3_:18%Yb^3+^,1%Er^3+^ core NPs, KMnF_3_:18%Yb^3+^,1%Er^3+^@KMnF_3_ core-inert-shell NPs and KMnF_3_:18%Yb^3+^,1%Er^3+^@KMnF_3_:2%Yb^3+^ core-active-shell NPs, we measured the XRD (X-Ray Diffraction) of these samples, and the results are shown in Figure 1. The diffraction peaks of the samples coincided well with the pure cubic-phase of KMnF_3_ (JCPDS-82-1334). The data suggests that the samples were pure cubic-phase KMnF_3_ NPs. In addition, the morphology of the above samples was analyzed by TEM. Figure 2 shows TEM images and size distributions of the core NPs, the core-inert-shell NPs, and the core-active-shell NPs, respectively. The result shows that the KMnF_3_ core NPs were cubic and monodisperse without any aggregate. The average diameter of the core NPs was around 23 ± 5 nm. When the core NPs were coated with an inert-shell or active-shell, KMnF_3_:18%Yb^3+^,1%Er^3+^@KMnF_3_ core-inert-shell NPs and KMnF_3_:18%Yb^3+^,1%Er^3+^@KMnF_3_:2%Yb^3+^ core-active-shell NPs were cubic, and the morphology of the two NPs did not change. The average sizes of the core-inert-shell NPs and the core-active-shell NPs were both about 65 ± 20 nm.

**Figure 3:**
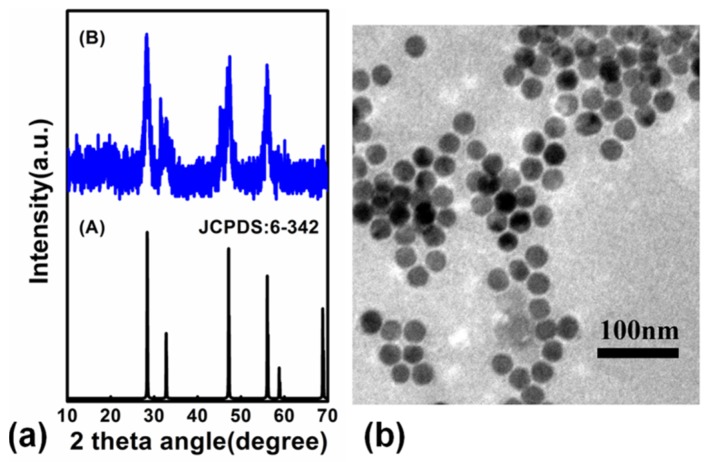
(**a**) XRD patterns of (**A**) standard NaYF_4_ NPs (JCPDS-6-342) and (**B**) NaYF_4_:18%Yb^3+^,1%Er^3+^ NPs; (**b**) TEM images of NaYF_4_:18%Yb^3+^,1%Er^3+^ NPs.

We also synthesized NaYF_4_:18%Yb^3+^,1%Er^3+^ NPs via a high-boiling solvent method. In order to get the phase of as-synthesized NaYF_4_:18%Yb^3+^,1%Er^3+^ NPs, we measured the XRD of the product, and the result is shown in Figure 3a. The diffraction peaks of the as-synthesized product coincided well with pure cubic-phase NaYF_4_ (JCPDS-6-342), and the results indicates that the products were pure cubic-phase NaYF_4_ NPs. Figure 3b shows the TEM of the NaYF_4_:18%Yb^3+^,1%Er^3+^ NPs. The data suggests that the product was spherical without agglomeration and the size of the as-synthesized product was 23 ± 3 nm.

### 3.2. Down-Shifting Luminescence Properties

**Figure 4:**
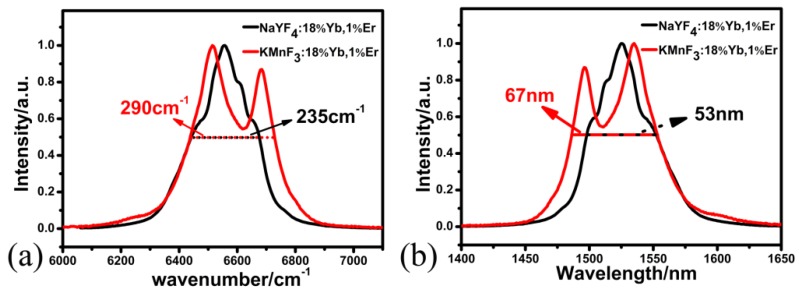
(**a**,**b**) Normalized down-shifting fluorescence spectra of NaYF_4_:18%Yb^3+^,1%Er^3+^ NPs and KMnF_3_:18%Yb^3+^,1%Er^3+^ NPs at the maximum value under diode laser excitation at 980 nm, where (**a**) the *x*-axis is wavenumber/cm^−1^ and (**b**) the *x*-axis is wavelength/nm.

In order to evaluate the impact of matrix material on the down-shifting fluorescence spectra (around 1.5 µm) of Yb^3+^-Er^3+^-co-doped fluoride nanoparticles, we compounded and characterized two NPs with the same size and crystal phase. Firstly, the NaYF_4_:18%Yb^3+^,1%Er^3+^ NPs and the KMnF_3_:18%Yb^3+^,1% Er^3+^ NPs were both cubic phase and had the same size. Then, the down-shifting fluorescence spectra of NaYF_4_:18%Yb^3+^,1%Er^3+^ NPs and KMnF_3_:18%Yb^3+^,1%Er^3+^ NPs were characterized. Figure 4 is the normalized down-shifting fluorescence spectra at maximum value of the two NPs excited by a 980-nm laser diode. From Figure 4, we can find the following: the down-shifting fluorescence spectra of KMnF_3_:Yb^3+^, Er^3+^ NPs had two emission peaks near the 1496 nm and 1534 nm bands, respectively, and the FWHM was 290 cm^−1^. The appearance of the two peaks of KMnF_3_:Yb^3+^,Er^3+^ NPs was due to stark splitting of the emitting level ^4^I_13/2_. In contrast, the down-shifting fluorescence spectra of NaYF_4_:Yb^3+^,Er^3+^ NPs had only one emission peak at 1525 nm, and the FWHM was 235 cm^-1^. The difference in down-shifting fluorescence spectra is attributed to differences in crystal filed in various host crystals. The FWHM of down-shifting fluorescence spectra of the KMnF_3_:Yb^3+^,Er^3+^ NPs was 55 cm^-1^ wider than that of NaYF_4_:Yb^3+^,Er^3+^ NPs. This means, when Er^3+^ and Yb^3+^ have the same concentration, the KMnF_3_:Yb^3+^,Er^3+^ NPs had a much wider FWHM of down-shifting fluorescence spectra than that of NaYF_4_:Yb^3+^,Er^3+^ NPs, with the same size and crystal phase, which shows the KMnF_3_:Yb^3+^,Er^3+^ NPs are a more suitable gain material for broadband waveguide amplifiers.

**Figure 5:**
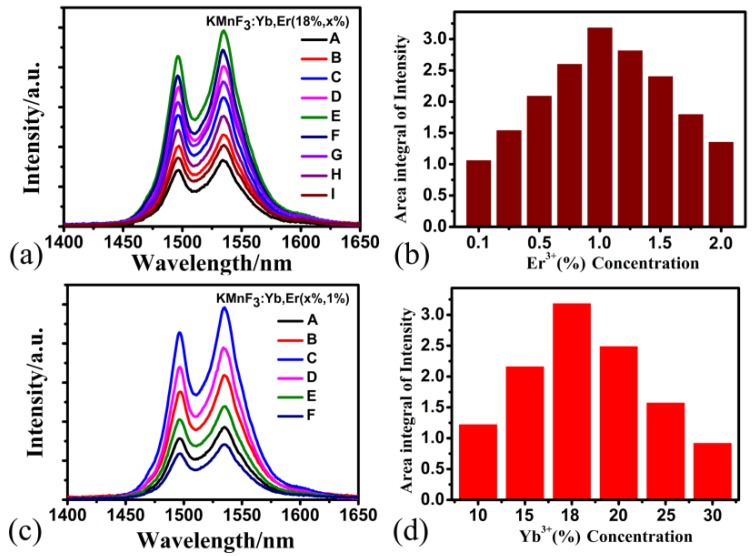
(**a**) Er^3+^ ion concentrations dependent down-shifting fluorescence of KMnF_3_:18%Yb^3+^,*x*%Er^3+^ (*x* = 0.1, 0.25, 0.5, 0.75, 1, 1.25, 1.5, 1.75, 2) NPs (curves A–I) under 980-nm laser excitation; (**b**) enhancement ratio of down-shifting emission dependent on Er^3+^ concentration of KMnF_3_:18%Yb^3+^,*x*%Er^3+^ NPs; (**c**) Yb^3+^ ion concentrations dependent down-shifting luminescence of KMnF_3_:*x*%Yb^3+^,1%Er^3+^ (*x* = 10, 15, 18, 20, 25, 30) NPs (curves A–F) under 980-nm laser excitation; (**d**) enhancement ratio of down-shifting emissions dependent on Er^3+^ concentrations of KMnF_3_:*x*%Yb^3+^,1%Er^3+^ NPs.

It is well known that the luminescence properties of Yb^3+^ and Er^3+^ co-doped fluoride nanoparticles can be influenced by the doping concentration of Yb^3+^ ions and Er^3+^ ions. In order to obtain the Yb^3+^ and Er^3+^ co-doped KMnF_3_ NPs with strong near-infrared emission at 1.5 μm, we synthesized a series of KMnF_3_ NPs with different concentrations of Yb^3+^ and Er^3+^ ions. Figure 5a shows the study of Er^3+^ ions concentration dependent down-shifting fluorescence of KMnF_3_ NPs and the down-shifting fluorescence of KMnF_3_:18%Yb^3+^,*x*%Er^3+^ (*x* = 0.1, 0.25, 0.5, 0.75, 1, 1.25, 1.5, 1.75, 2) NPs under diode-laser excitation at 980 nm. Figure 5b shows the variation of fluorescence intensity of KMnF_3_ NPs and concentration of Er^3+^ ions. We can see that when the concentration of Er^3+^ ions is 1%, the down-shifting luminescence intensity of KMnF_3_ NPs gets its maximum value, and when the concentration of Er^3+^ ions is lower or higher than 1%, the luminescence intensity is lower than the maximum intensity. The experimental results shown in Figure 5b indicate that the optimum concentration of Er^3+^ ions doped with KMnF_3_:18%Yb^3+^,*x*%Er^3+^ NPs is 1%.

At the same time, we also investigated the effect of Yb^3+^ ions on the fluorescence intensity of KMnF_3_ NPs. Figure 5c shows the down-shifting fluorescence spectra of KMnF_3_:*x*%Yb^3+^,1%Er^3+^ NPs excited by a 980 nm laser diode. We can see from Figure 5d that when the concentration of Yb^3+^ ions is 18%, the down-shifting fluorescence intensity of KMnF_3_ NPs obtains its maximum value, which indicates that the optimum concentration of Yb^3+^ ions doped with KMnF_3_: *x*%Yb^3+^,1%Er^3+^ NPs is 18%.

**Figure 6:**
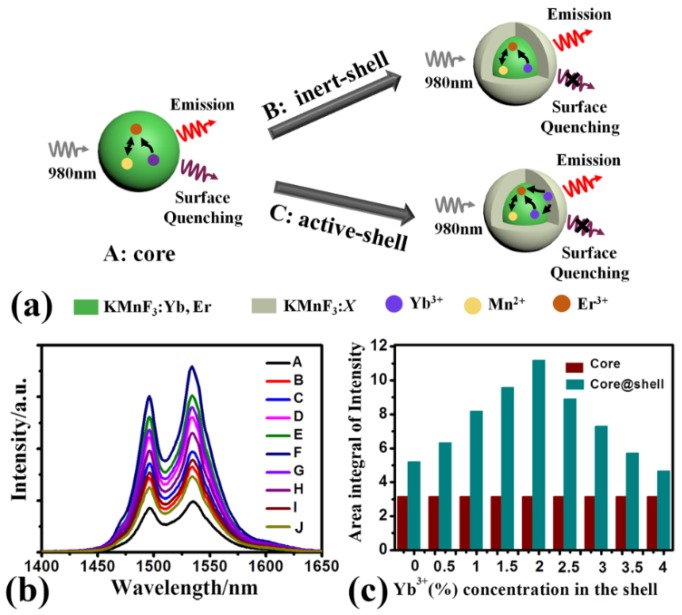
(**a**) Schematic illustration of KMnF_3_:18%Yb^3+^,1%Er^3+^ NPs, KMnF_3_:18%Yb^3+^,1%Er^3+^@KMnF_3_ NPs, and KMnF_3_:18%Yb^3+^,1%Er^3+^@KMnF_3_:2%Yb^3+^ NPs. (**b**) Down-shifting luminescence of KMnF_3_:18%Yb^3+^,1%Er^3+^ NPs (curve A) and KMnF_3_:18%Yb^3+^,1%Er^3+^@KMnF_3_:*x*%Yb^3+^ (*x* = 0, 0.5, 1, 1.5, 2, 2.5, 3, 3.5, 4) NPs (curve B–J). (**c**) Enhancement ratio of down-shifting emissions dependent on Yb^3+^ concentrations of NPs.

In this part, we demonstrate and analyze the effect of Yb^3+^ concentrations in the shell on the down-shifting fluorescence intensity of KMnF_3_ core-shell NPs. Figure 6a shows the schematic illustration of KMnF_3_:18%Yb^3+^,1%Er^3+^ NPs, KMnF_3_:18%Yb^3+^,1%Er^3+^@KMnF_3_ NPs, and KMnF_3_:18%Yb^3+^,1%Er^3+^@KMnF_3_:2%Yb^3+^ NPs. The down-shifting fluorescence spectra and its corresponding enhancement ratio of KMnF_3_ core NPs and a series of KMnF_3_ core-shell NPs with different Yb^3+^ concentrations in the shell are shown in Figure 6b,c, respectively. In Figure 6b, the down-shifting fluorescence spectrum corresponding to curve A indicates the KMnF_3_:18%Yb^3+^,1%Er^3+^ core NPs had the weakest near-infrared emission from ^4^I_13/2_ → ^4^I_15/2_ transition of Er^3+^ ions. The reason is because the high-specific surface area of the KMnF_3_:Yb^3+^,Er^3+^ core NPs led to a large number of surface defects which can quench the energy of pump light. After the core NPs were coated by inert shells (the shell without Yb^3+^ ions), which can limit the efficiency of surface quenching [26,32,33], the down-shifting fluorescence intensity of KMnF_3_:18%Yb^3+^,1%Er^3+^@KMnF_3_ core-inert-shell NPs increased. As shown in Figure 6c, the down-shifting fluorescence intensity of the KMnF_3_:Yb^3+^,Er^3+^ core-inert-shell NPs was 1.6 times that of KMnF_3_:Yb^3+^,Er^3+^ core NPs. Then, the core NPs were coated by active shells which were doped with Yb^3+^ ions. The intensity of the fluorescence spectrum of the KMnF_3_:Yb^3+^,Er^3+^@KMnF_3_:*x*%Yb^3+^ (*x* = 0.5, 1, 1.5, 2, 2.5, 3, 3.5, 4) core-active-shell NPs are shown by curves C–J in Figure 6b. From the enhancement ratio of the KMnF_3_ core-active-shell NPs with different Yb^3+^ concentrations shown in Figure 6c, we can see that when the concentration of Yb^3+^ is 2%, the down-shifting luminescence intensity of the core-active-shell NPs reaches its maximum value which was 2.1 times that of the core-inert-shell NPs under 980 nm excitation. The reason is that the KMnF_3_:2%Yb^3+^ active shell can transfer the energy of pump light to the core region efficiently, while overcoming surface quenching effect [34,35,36,37]. In addition, we measured the fluorescent decay curves of the ^4^I_13/2_ level of Er^3+^ ions in KMnF_3_:18%Yb^3+^,1%Er^3+^ core NPs, KMnF_3_:18%Yb^3+^,1%Er^3+^@KMnF_3_ core-inert-shell NPs, and KMnF_3_:18%Yb^3+^,1%Er^3+^@KMnF_3_:2%Yb^3+^ core-active-shell NPs, as shown in Figure S2. The data agrees well with the intensity of these NPs. We measured the down-shifting fluorescence of KMnF_3_:18%Yb^3+^,1%Er^3+^@KMnF_3_:2%Yb^3+^ core-active-shell NPs was excited by different power of 980 nm, as shown in Figure S3. The experimental results indicate that the KMnF_3_:18%Yb^3+^,1%Er^3+^@ KMnF_3_:2%Yb^3+^ core-active-shell NPs with strong broadband down-shifting luminescence has great advantages as gain medium for waveguide amplifiers.

### 3.3. Optical Waveguide Amplifiers Based on KMnF_3_:18%Yb^3+^,1%Er^3+^@KMnF_3_:2%Yb^3+^ NPs

**Figure 7:**
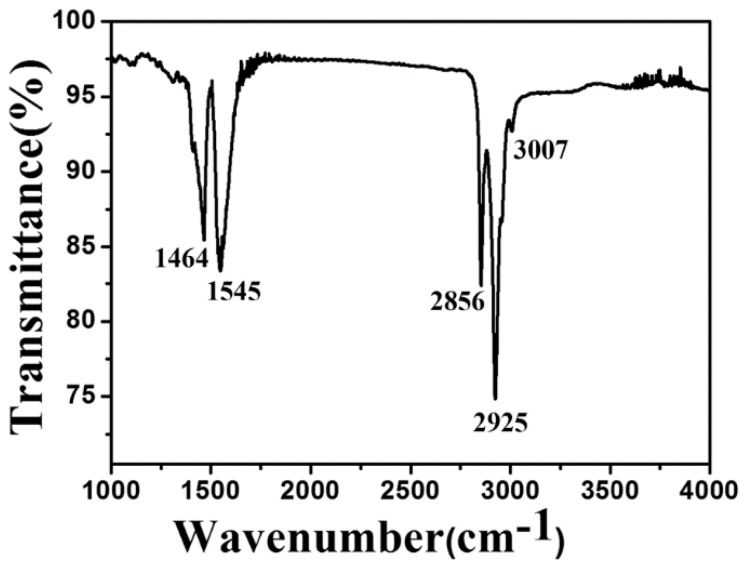
The FTIR spectrum of the oleic-acid-coated KMnF_3_:18%Yb^3+^,1%Er^3+^@KMnF_3_:2%Yb^3+^ NPs.

A FTIR spectrum of the KMnF_3_:18%Yb^3+^,1%Er^3+^@KMnF_3_:2%Yb^3+^ core-active-shell NPs is shown in Figure 7. The absorptions at around 3007 cm^−1^ was due to the symmetric stretching vibration of the =C–H group. The peaks located at 2856 cm^−1^ and 2925 cm^−1^ can be assigned as the symmetric and asymmetric vibration of the –CH_2_ group. The absorptions at 1545 cm^−1^ and 1464 cm^−1^ are attributed to the asymmetric and symmetric vibration of the group (–COOH) of the oleic acid coating. As the above result shows, that core-active-shell NPs were coated by the oleic acid ligand. Then, the KMnF_3_:18%Yb^3+^,1%Er^3+^@KMnF_3_:2%Yb^3+^ core-active-shell NPs were dispersed into PMMA as the gain medium of polymer waveguides.

**Figure 8:**
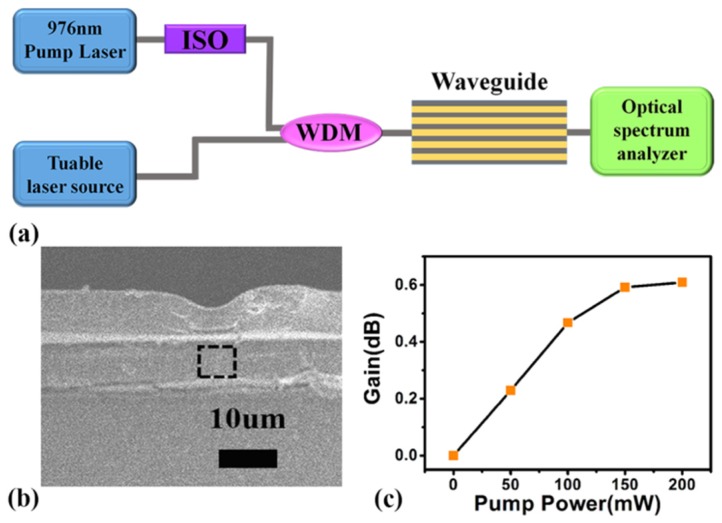
(**a**) Schematic illustration of measuring the optical gain of waveguide amplifiers. (**b**) SEM image of the KMnF_3_:18%Yb^3+^,1%Er^3+^@KMnF_3_:2%Yb^3+^ NPs doped with polymer waveguide. (**c**) The relative gain as a function of pump power (980 nm) with different input signal powers (1534 nm) in KMnF_3_:18%Yb^3+^ ,1%Er^3+^@KMnF_3_:2%Yb^3+^ NPs doped with polymer waveguide.

We manufactured polymer waveguide amplifiers whose gain medium was made by mixing KMnF_3_:18%Yb^3+^,1%Er^3+^@KMnF_3_:2%Yb^3+^ with strong near-infrared luminescence and PMMA evenly. The schematic of our polymer waveguide amplifier gain testing system is shown in Figure 8a. In our gain testing system, a 980-nm laser and a tunable laser source (Santec TSL-210, Aichi, Japan) were separately used as pump light and signal light, where the wavelength range of Santec TSL-210 was 1510 nm to 1590 nm. After the pump light passed through the isolator (ISO), a wavelength division multiplexer (WDM) was used to couple the ISO’s output and the signal light into an optical fiber whose output light passed through an optical waveguide amplifier. The output of the optical waveguide amplifier was monitored by the Ando AQ-6315A spectrometer (optical spectrum analyzer, OSA). The morphology of the cross-section of the KMnF_3_:18%Yb^3+^,1%Er^3+^@KMnF_3_:2%Yb^3+^ NPs doped waveguide is displayed by Figure 8b. The following equation can be used to calculate the relative gain of our waveguide amplifiers [19].

$$Gain[dB]=10log\left(\frac{P_{s-out}^{p}}{P_{s-out}}\right)=10log(P_{s-out}^{p})-10log(P_{s-out})$$

where $P_{s-out}^{p}$ and $P_{s-out}$ represent the output signal powers in cases with and without pump light [19], respectively. Figure 8c shows the curve of relative gain versus pump power at 980 nm of a 10 mm-long waveguide, when the input signal power and wavelength are 0.2 mW and 1534 nm, respectively. As Figure 8c shows, when the pump power is in the range of 0 mW to 150 mW, the relative gain increases rapidly with the increase of the pump power. When the pump power is 200 mW, the maximum relative gain of 0.6 dB is obtained.

In this paper, we synthesized KMnF_3_:18%Yb^3+^,1%Er^3+^@KMnF_3_:2%Yb^3+^ NPs with broadband near-infrared emissions at 1.5 µm and constructed KMnF_3_:18%Yb^3+^,1%Er^3+^@KMnF_3_:2%Yb^3+^ core-active-shell NPs doped with polymer waveguides, but we did not obtain broadband optical gain. It is clear that the luminescence intensity of the core-active-shell NPs and fabrication method of the optical waveguides would affect the testing result. A study on the above factors affecting the optical gain of the waveguide amplifiers is in progress and will be the subject of a future paper.

## 4. Conclusions

In conclusion, we synthesized KMnF_3_:18%Yb^3+^,1%Er^3+^ NPs with a broadband down-shifting emissions under 980 nm of excitation, and the FWHM of the down-shifting luminescence spectra of this NPs was 290 cm^−1^. The KMnF_3_:18%Yb^3+^,1%Er^3+^@KMnF_3_:2%Yb^3+^ NPs was prepared by coating a KMnF_3_:2%Yb^3+^ active-shell on KMnF_3_:Yb^3+^,Er^3+^ core NPs. The intensity of the core NPs was enhanced 3.4 times after coating a KMnF_3_:2%Yb^3+^ active-shell. We manufactured polymer waveguide amplifiers by using KMnF_3_:18%Yb^3+^,1%Er^3+^@KMnF_3_:2%Yb^3+^ NPs with PMMA as the gain medium. The relative optical gain of our wave guide amplifiers was 0.6 dB, when the input signal power and pump power were 0.2 mW and 200 mW, respectively.

## Supplementary Materials

The following are available online at https://www.mdpi.com/2079-4991/9/3/463/s1, Figure S1: Schematic illustration of fabrication processes of the KMnF_3_:18%Yb^3+^,1%Er^3+^@KMnF_3_:2%Yb^3+^ core-active-shell NPs-doped polymer waveguides. Figure S2: The fluorescent decay curves of the ^4^I_13/2_ level of Er^3+^ ions in KMnF_3_:18%Yb^3+^,1%Er^3+^ core NPs, KMnF_3_:18%Yb^3+^,1%Er^3+^@KMnF_3_ core-inert-shell NPs and KMnF_3_:18%Yb^3+^,1%Er^3+^@KMnF_3_:2%Yb^3+^ core-active-shell NPs. Figure S3: (a) The down-shifting fluorescence of KMnF_3_:18%Yb^3+^,1%Er^3+^@KMnF_3_:2%Yb^3+^ core-active-shell NPs was excited by different power of 980 nm. (b) The relationship between luminous intensity and pump power.
