# Supplementary material for: KMnF3:Yb3+,Er3+ Core-Active-Shell Nanoparticles with Broadband Down-Shifting Luminescence at 1.5 μm for Polymer-Based Waveguide Amplifiers
Source: Nanomaterials (Basel). 2019 Mar 20;9(3):463. doi: 10.3390/nano9030463 (PMC6474087; doi:10.3390/nano9030463)
Supplement: Supplementary file 1 [file nanomaterials-09-00463-s001.pdf]

nanomaterials

Supplementary Materials:

$KMnF_3:Yb^{3+},Er^{3+}$ Core-Active-Shell Nanoparticles with Broadband Down-shifting Luminescence at 1.5 μm for Polymer-based Waveguide Amplifiers

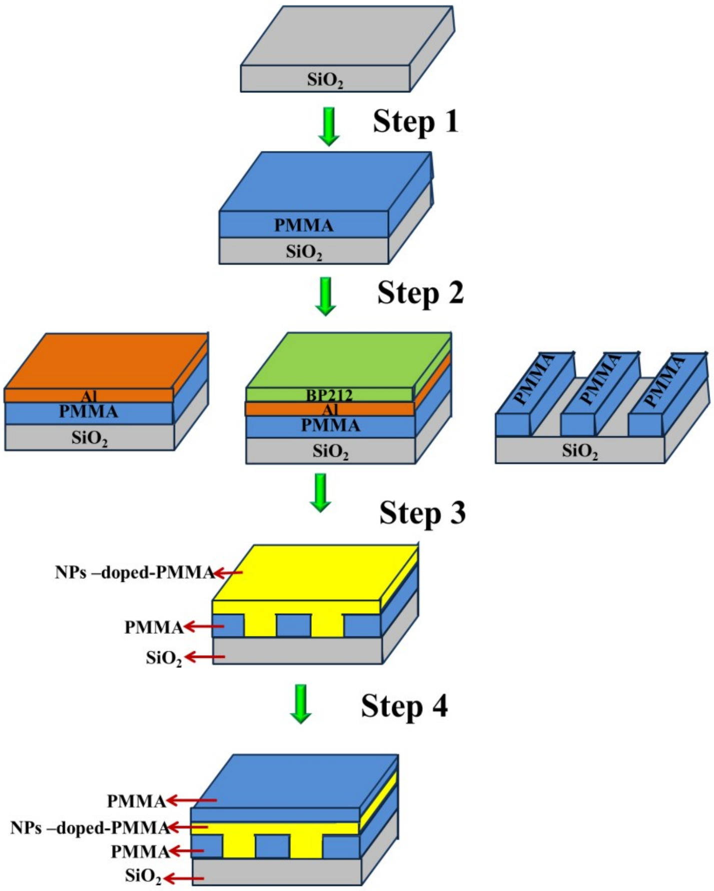

Figure S1. Schematic illustration of fabrication processes of the $KMnF_3$:18%Yb^{3+},1%Er^{3+}@$KMnF_3$:2%Yb^{3+} core-active-shell NPs-doped polymer waveguides.

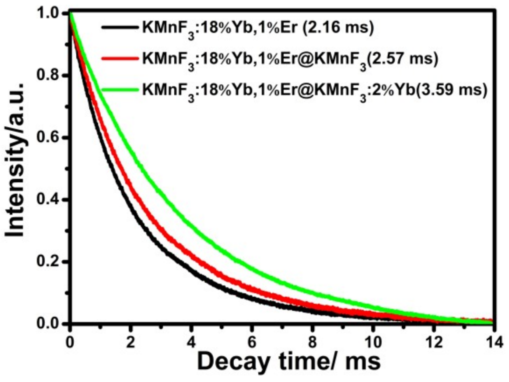

Figure S2. The fluorescent decay curves of the $^4I_{13/2}$ level of Er^{3+} ions in $KMnF_3$:18%Yb^{3+},1%Er^{3+} core NPs, $KMnF_3$:18%Yb^{3+},1%Er^{3+}@$KMnF_3$ core-inert-shell NPs and $KMnF_3$:18%Yb^{3+},1%Er^{3+}@$KMnF_3$:2%Yb^{3+} core-active-shell NPs.

Figure S2 show the fluorescence lifetime of the $^4I_{13/2}$ level of Er^{3+} ions in $KMnF_3$:18%Yb^{3+},1%Er^{3+} core NPs, $KMnF_3$:18%Yb^{3+},1%Er^{3+}@$KMnF_3$ core-inert-shell NPs and $KMnF_3$:18%Yb^{3+},1%Er^{3+}@$KMnF_3$:2%Yb^{3+} core-active-shell NPs by using a 980 nm pulsed laser with a pulse width of 30 ns and a repetition rate of 50 Hz as the excitation source. The results show that the lifetime of the $^4I_{13/2}$ level of Er^{3+} in $KMnF_3$:18%Yb^{3+},1%Er^{3+} core NPs is 2.16 ms. When coating a $KMnF_3$ inert-shell (There is no Yb^{3+} ions in the shell) on the core NPs, the lifetime of the $^4I_{13/2}$ level of Er^{3+} in $KMnF_3$:18%Yb^{3+},1%Er^{3+}@$KMnF_3$ core-inert-shell NPs was increased to 2.57 ms. When the shell was introduced 2%Yb^{3+} ions, the lifetime of the $^4I_{13/2}$ level of Er^{3+} in $KMnF_3$:18%Yb^{3+},1%Er^{3+}@$KMnF_3$:2%Yb^{3+} core-active-shell NPs was increased to 3.59 ms.

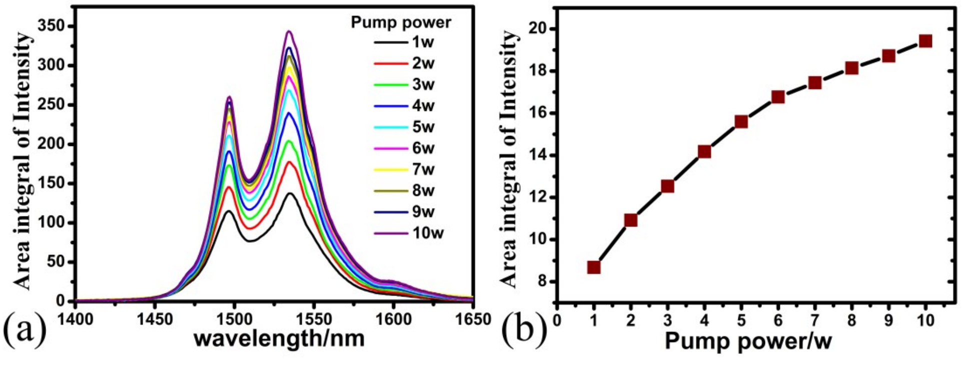

Figure S3. (a) The down-shifting fluorescence of $KMnF_3$:18%Yb^{3+},1%Er^{3+}@$KMnF_3$:2%Yb^{3+} core-active-shell NPs was excited by different power of 980 nm. (b) The relationship between luminous intensity and pump power.

From the Figure S3, you can see that with the increase of pump power, the down-shifting fluorescence increases gradually.
